# The influence of methylphenidate on auditory brainstem response patients with attention deficit hyperactivity disorder; an exploratory study

**DOI:** 10.1177/22799036231204319

**Published:** 2023-10-11

**Authors:** Emma Claesdotter-Knutsson, Johan Källstrand, Katalin Niklasson, Mitchell Andersson, Magnus Lindvall

**Affiliations:** 1 Psychiatry, Department of Clinical Sciences Lund, Faculty of Medicine, Lund University, Lund, Sweden; 2 Independent Researcher; 3 Outpatient Department, Child and Adolescent Psychiatry Clinic, Lund, Sweden

**Keywords:** ABR, ADHD, child and adolescent psychiatry, methylphenidate, thalamus

## Abstract

**Background:**

Attention-deficit hyperactivity disorder (ADHD), characterized by periods of inattention, overactivity, and impulsiveness, is the most prevalent neurodevelopmental disorder among children. Auditory Brainstem Response (ABR) is a technique in which clickshaped sounds elicit potentials that are recorded from electrodes placed on a patient’s skull. Extant research indicates that ABR is frequently affected in neurodevelopmental disorders such as ADHD. Methylphenidate (MPH), a psychostimulant, is often prescribed to children with ADHD as a first-line pharmacological treatment. The aim of this study was to explore the effects of Methylphenidate treatment on previously observed amplitude alterations in the ABR of patients with ADHD.

**Methods:**

We recruited 32 drug-naïve children and adolescents (19 males and 13 females; mean age 11 years) diagnosed with ADHD and 35 health controls (15 males and 20 females; mean age 12 years). The ADHD group was treated with Methylphenidate, and ABR was recorded before treatment and at a steady state of medical treatment.

**Results:**

Medicated ADHD patients exhibited increased activity in the right side ABR in Wave VI.

**Conclusions:**

A significant increase in activity was found in a part of the ABR thought to correspond to the thalamic area in medicated ADHD patients compared to the same area of non-medicated ADHD patients. The results add to the growing body of research suggesting that specific ABR peaks correlate to certain psychiatric symptoms.

## Introduction

Attention deficit hyperactivity disorder (ADHD) is a common neurodevelopmental disorder with an estimated 5% global prevalence in children.^1–3^ ADHD is defined by age inappropriate and persisting periods of inattention, overactivity, and/or impulsiveness.^4,5^ ADHD is associated with an increased risk of academic failure, mental illness, and delinquency, as well as a significant burden on the health, social care, and criminal justice systems.^4,6^

**Figure 1.**
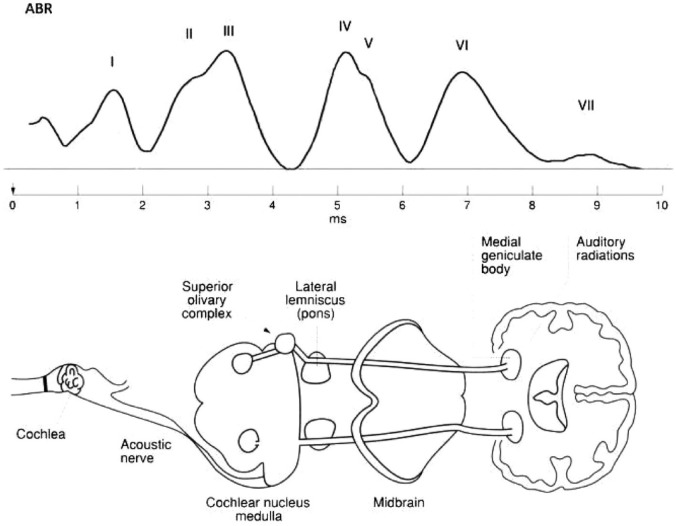
The standard ABR and corresponding anatomical structures.

Electroencephalography (EEG) recordings of neural activity in response to a specific, repeated stimulus, are known as Event Related Potentials (ERP). The ERP is known as Auditory Brainstem Response (ABR) when sound is the stimulus of choice. ABR has been used by pediatricians to diagnose possible hearing defects since the 1970s.^7^ ABR traditionally uses click sounds at predetermined intervals and calibrated electrodes placed on the skull to measure the processing that occurs in the basic auditory pathways situated in the brainstem in order to avoid recording later responses from cerebral clusters of higher processing complexity. Using complex stimuli, as in forward and backward masking, the sounds in ABR are similar to sounds in everyday hearing.^8^ Complex stimuli may reveal aberrations that would be missed by standard audiological ABR procedures.^9–12^ The ABR consists of seven positive peaks (Wave I–VII) recorded by surface electrodes on the mastoid processes of each ear and the forehead.^7^ Previous research indicates that Waves III are generated in the auditory nerve, Wave III in the cochlear nucleus, Waves IV and V in the mid-brain, and Waves VI and VII in the thalamus, specifically the medial geniculate body (MGB)^13^ (Figure 1).

Based on research indicating that specific ABR peaks are correlated with certain psychiatric symptoms, it has been speculated that ABR could be used as a complementary clinical tool to aid in the diagnosis of schizophrenia,^10,14^ bipolar disorders,^12^ autism spectrum disorders,^15–17^ and ADHD.^11,18,19^

Methylphenidate (MPH) is a psychostimulant commonly prescribed as a first-line therapy for pediatric ADHD.^20,21^ ADHD symptoms are believed to partly emerge from catecholamine neurotransmission dysfunction predominantly in the prefrontal cortex.^22^ MPH produces its therapeutic effects by elevating the levels of extracellular synaptic dopamine (DA) and norepinephrine (NE) via reuptake transporter inhibition and vesicular monoamine transporter 2 (VMAT-2) redistribution in the central nervous system. MPH also functions as an agonist at the serotonin 1A receptor (5-HT1A), which partially regulates dopamine release.^22,23^ MPH-related enhanced DA and NE neurotransmission is hypothesized to engage selfcontrol regulation networks, reducing the severity of ADHD symptoms.^24,25^ There is very little research on ABR and ADHD in children but even less on the potential effect of MPH on ABR. To our knowledge Jonkman et al. are the only ones that have studied the possible effect of MPH on ABR.^25^ Jonkman et al. showed that MPH normalized ABR amplitudes in children with ADHD suggesting that its effects might be established through influences on the posterior (parietal) cortex.^25^

Our aim in this exploratory study is to compare the peak ABR amplitudes corresponding to Waves I-VI (P1-P6) before and during MPH treatment in children with ADHD.

## Materials and methods

### Subjects

This study was conducted April to September in 2021 it included 32 drug-naïve patients with ADHD (*Mean* Age = 11 ± 2.6, 41% female) and 35 HC (*Mean* Age = 12 ± 2.6, 57% female). The ADHD group was treated with MPH, and ABR was recorded at baseline and after one month during which they had been titrated up to methylphenidate 36 mg. Nine patients chose to withdraw from the study before the second ABR, leaving a total of 23 patients (*Mean* Age = 12 ± 2.3, 52% female ).

All patients were recruited from the child and adolescent outpatient clinic in Lund, a city in the south of Sweden. All patients were diagnosed according to the Diagnostic and Statistical Manual of Mental Disorders, 5th Edition (DSM-5; APA 2013). A senior child and adolescent psychiatrist confirmed all ADHD diagnoses.

Patients with concurrent psychiatry diagnoses including intellectual disability and hearing impairments verified through interviews with parents were excluded from the study. All patients were drug naïve.

Patients were instructed not to have any intake of activating substances such as energy drinks, Coca-Cola, or coffee prior to the tests.

Written informed consent was obtained from all the subjects and their parents/guardians. The study was approved by the regional ethics committee at Lund University (Dnr: 2021-00351).

### Apparatus

SensoDetect BERA (Brainstem Evoked Response Audiometry) A1000 was used to record ABRs. Stimuli were presented binaurally in phase via TDH-50 P headphones with Model 51 cushions (Telephonics, Farmingdale, New York, USA). Click pulses were presented until 1024 accepted evoked potentials were obtained for each stimulus. Recording sweeps were coordinated with the auditory stimuli using transistor-transistor logic (TTL) trigger pulses.

Stimuli sound levels were calibrated with a Bruel & Kjaer 2203 sound level meter and type 4152 artificial ear (Bruel & Kjaer S & VMeasurement, Naerum, Denmark). The acoustic output power of stimuli measured from the headphones corresponded to SPL: 80 dB HL or 109 peSPL (peak equivalent). All recorded data for each sound stimulus from each ear of every study participant were imported into Microsoft Excel (Microsoft Corp, Redmond, WA, USA) for statistical analysis.

A square-shaped click pulse was used as a probe for both the standard ABR stimulus and the auditory forward masking condition. The total duration time of each probe was 0.136 ms with a rise and fall time of 0.023 ms and had an interstimulus interval (ISI) of 0.192 s. A 1500 Hz low pass filtered noise (Butterworth filter) with a duration of the 0.015 s including a 0.004 s rise and fall time was used as masker. The masker and target probe were separated with a 0.012 s gap.

The stimuli were created using MATLAB Signal Processing Toolbox (The MathWorks, Inc., Natick, Massachusetts, USA) and stored in flash memory in the SensoDetect® BERA system.

### Procedure

Participants were seated in an armchair in a resting position. The tests were performed in a quiet and darkened room with Farraday’s cage put around the patient to avoid electrical interference.^26^ Surface electrodes were attached to the skin over the mastoid bones behind the left and right ear. A ground electrode and a reference electrode were placed on the vertex and forehead, respectively. Before presenting the auditory stimuli, the procedure was fully explained to the test subject. The click stimuli were presented beforehand to acquaint the participant with the sounds. Before and after the experiments, absolute impedances and interelectrode impedance were measured to ascertain that electrode contact was maintained below 5000 Ω. The subjects were instructed to close their eyes and to relax. Test persons were permitted to fall asleep during the test as no active participation was needed. The subjects were tested one at a time with a total duration of 15 min. At the time for the second ABR the patients were all at a dose of 36 mg MPH.

### Data analysis

The ABR curve quality check was measured through whole curve correlation (Spearman’s ρ, 0–10 MS) between the specific patient and the norm ABR curve. The norm ABR curve consisted of the median representation from a group of normal hearing healthy controls. This method is a standard operating procedure to grant ABR quality. Collected evoked potentials for each sound stimulus from each individual were imported to Microsoft Excel (Microsoft Corp, Redmond, WA, USA) and analyzed using SensoDetect® BAS. We studied the peak-totrough amplitudes in six peaks (P1, P2, P3, P4, P5, P6). For each subject, amplitudes for left and right were identified for the square-shaped click as the target stimulus preceded by a masker of 70 dB (FM). The amplitudes were calculated by subtracting the minimum value from the preceding max value in the peak-to-trough area for each peak, as defined by the healthy control group average curves. Four ABR waveforms were obtained from each test subject (two left and two right) and 20 values. The nonparametric Mann–Whitney *U* test was used to measure statistical differences.

## Results

We studied the ABR under forward masked condition looking at the peak-to-trough amplitudes of the characterized peaks in the brainstem audiograms for both ears. We looked at values for five peaks: P1, P2, P3, P5, and P6. P4 was excluded due to missing values. Due to drop out, it was only possible to collect data on 23 of the 32 medicated patients with ADHD. We found that P6 peak-to-troughs were significantly greater on the right side for ADHD subjects compared to the unmedicated ADHD subjects (Table 1), *p* = 0.008, *z* = −2.414.

**Table 1.** Peak to trough amplitudes for five ABR peaks, ADHD patients unmedicated versus medicated.

	Left side ABR			Right side ABR		
	ADHD (*n* = 32), µV		ADHD medicate (*n* = 23), µV	ADHD (*n* = 32), µV		ADHD medicate (*n* = 23), µV
P1	0.29	n.s.	0.33	0.28	n.s.	0.31
P2	0.2	n.s.	0.19	0.14	n.s.	0.14
P3	0.41	n.s.	0.34	0.35	n.s.	0.36
P5	0.27	n.s.	0.28	0.31	n.s.	0.31
P6	0.27	n.s.	0.29	0.23	0,00788	0.27

## Discussion

**Figure 2.**
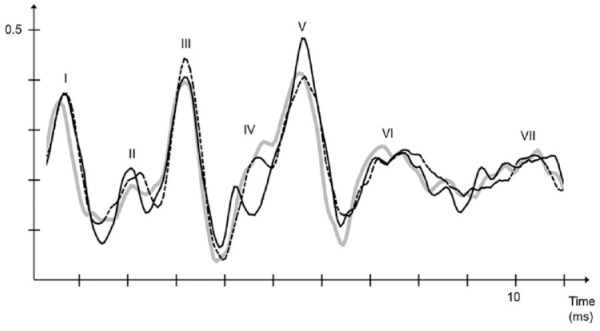
Figure: healthy controls (grey line), never medicated ADHD (dotted black line) and ADHD medicated with MPH (solid black line).

The aim of this study was to explore possible alterations in the ABR after administration of MPH among 32 drug naïve ADHD patients. We found a significant increase in activity in a part of the ABR that is believed to correspond to the right thalamic area in the medicated patients with ADHD. This is in line with MRI studies, showing both under-active thalamic activity in ADHD and upregulated thalamic activity after MPH intake.^27^ To further substantialize our findings, we compared our data to age- and gender-matched healthy controls (Table 2). We found the tendency for ABR to normalize when patients were medicated, however, P6 waves generated from the right side remained significantly elevated above baseline (Figure 2).

**Table 2.** Peak to trough amplitudes for five ABR peaks, HC versus ADHD patients unmedicated versus medicated.

	Left side ABR					Right side ABR				
	HC (*n* = 35), µV		ADHD (*n* = 32), µV		ADHD medicate (*n* = 23), µV	HC (*n* = 35), µV		ADHD (*n* = 32) µV		ADHD medicate (*n* = 23), µV
P1	0.33	n.s.	0.29	n.s.	0.33	0.26	n.s.	0.28	n.s.	0.31
P2	0.18	n.s.	0.2	n.s.	0.19	0.15	n.s.	0.14	n.s.	0.14
P3	0.38	n.s.	0.41	n.s.	0.34	0.35	n.s.	0.35	n.s.	0.36
P5	0.36	n.s.	0.27	n.s.	0.28	0.31	n.s.	0.31	n.s.	0.31
P6	0.32	0.005	0.27	n.s.	0.29	0.29	0.045	0.23	0.008	0.27

The role of the thalamus is thought to be prominent in cases of ADHD. Previous studies^28,29^ have described that the thalamus acts as a relay between various subcortical areas and the cerebral cortex. Furthermore, several studies have reported that the activity in the thalamus results in brainwaves detected in EEG and ABR.^30–33^ However, the specificity of ABR findings linked to ADHD have lately been called into question, for example, by Talge and colleagues (2021) who studied ABR latencies, are advocating for more research into cortico-striato-thalamo-cortical (CSTC) loops.^34^ Our findings should be looked upon in relation to other neuropsychiatric groups such as autism spectrum disorder. Our findings are well in line with research^35^ showing that the thalamus is key in hyperarousal and increased alertness, a pervasive problem for children with ADHD. Positive effects of MPH on ABR amplitudes are thought to be mediated by NE projecting from locus Coeruleus via Thalamus to the cortex.^22,25^ This pathway is important for attention processing.^22,25,34^

Our results add to the growing body of research suggesting that specific ABR peaks correlate to certain psychiatric symptoms. The paradigm built on this hypothesis has previously even led to suggestions that ABR could be used as a complementary diagnostic tool in psychiatric settings and our findings contribute to the evidence supporting the potential of ABR as a complementary diagnostic tool in the future.

### Limitations

Limitations of this study include that the results are based on group differences. It is well known that there are gender-based differences in ADHD and the overrepresentation of male ADHD subjects might have influenced the result. Furthermore, our study has a restricted age range, leading to limited generalizability to older populations. An additional source of potential bias is a relatively small sample size and a loss to follow-up. Nine of the participants withdrew from the study leading to missing observations that may lead to bias in the effect estimate. The primary reason for loss to follow-up was the gap in time between study visits (≥1 month). Lastly, the present study’s hypotheses and interpretations are based on a reductionistic approach to psychiatry positing that higher order mental disorders (e.g., ADHD) are the result of lower order biological abnormalities (i.e., abnormal P6 amplitudes).

The authors acknowledge that this approach may bias our interpretations.^36^

The significance of right-side aberrations in ABRs should be further investigated. As the number of participants where limited, further studies with more patients could clarify the impact of MPH on ABRs in ADHD pediatric patients

Future research could investigate whether other ADHD medications also have an impact on ABRs. In addition, longitudinal studies are needed to evaluate the causal link between psychostimulant medication use, ADHD, and ABRs.
